# Supplementary material for: Heat adaptation of phage T7 under an extended genetic code
Source: Virus Evol. 2021 Dec 1;7(2):veab100. doi: 10.1093/ve/veab100 (PMC8923235; doi:10.1093/ve/veab100)
Supplement: veab100_Supp [file veab100_supp.zip › Sequencing_Analysis.zip!/Sequencing_Analysis/A1/evidence/JC_SIDE_1_62.html]

BRESEQ :: Evidence


| New junction evidence | | | | | | | | | | | |
| --- | --- | --- | --- | --- | --- | --- | --- | --- | --- | --- | --- |
|  | | seq id | position | reads (cov) | reads (cov) | score | skew | freq | annotation | gene | product |
| \* | ? | NC\_001604.1 | = 13586 | 3 (0.000) | 2345 (0.770) | 347/592 | 0.3 | 93.9% | intergenic (–/–) | *–/–* | –/– |
|  | ? | NC\_001604.1 | 14356 = | 301 (0.100) |  |  |  |  | intergenic (–/–) | *–/–* | –/– |

Only 100 of 2370 total aligned reads displayed.  

`CAGACGACTACGAGAAGTTTCGCTGGAGAGTCCCATTCTAATACGACTCACTAAAGGAGACACACCATGTTCAAACTGATTAAGAAGTTAGGCCAACTGCTGGTTCGTATGTACAACGTGGAAGCCAAGCGACTGAACGATGAGGCTCGTAAAGAGGCCACACAGTCACGCGCTCTGGCGATTCGCTCCAACGAACTGGCTGACAGTGCATCCACTAAAGTTACCGAGGCTGCCCGTGTGGCAAACCAAGCTCAACAGCTTTCCAAATTCTTTGAGTAATCAAACAGGAGAAACCATTATGTCTAACGTAGCTGAAACTATCCGTCTATCCGATACAGCTGACCAGTGGAACCGTCGAGTCCACATCAACGTTCGCAACGGTAAGGCGACTATGGTTTACCGCTGGAAGGACTCTAAGTCCTCTAAGAATCACACTCAGCGTATGACGTTGACAGATGAGCAAGCACTGCGTCTGGTCAATGCGCTTACCAAAGCTGCCGTGACAGCAATTCATGAAGCTGGTCGCGTCAATGAAGCTATGGCTATCCTCGACAAGATTGATAACTAAGAGTGGTATCCTCAAGGTCGCCAAAGTG  >  NC_001604.1/13286‑13881`  

                                                                                                                                                                                                                                                                                                            `|`                                                                                                                                                                                                                                                                                                         
  `gacgacTACGAGAAGTTTCGCTGGAGAGTCCCATTCTAATACGACTCACTAAAGGAGACACACCATGTTCAAACTGATTAAGAAGTTAGGCAAACTGCTGGTTCGTATGGACAACGTGGAAGCCAAGCGACTGAACGATGAGGCTCGTAAAGAGGCCACACAGTCACGCGCTCTGGCGATTCGCTCCAACGAACTGGCTGACAGTGCATCCACTAAAGTTACCGAGGCTGCCCGTGTGGCAAACCAAGCTCAACAGCTTTCCAAATTCTTTGAGTAATCAAACAGGAGAAACCATTATGat                                                                                                                                                                                                                                                                                                       <  1:341890‑M1/301‑3 (MQ=255)`  
  `aacgacaACGAGAAGTTTCGCTGGAGAGTCACATTCTAATACGACTCACTAAAGGAGACACACCATGTTCAAACAGATTAAGAAATTAGGCCAACTGCTGGTTCGTATGTACAACGTGGAAGCCAAGCGACTGAACAATGAGGCTCGTAAAGAGGCCACACAGTCACGCGCTCTGGCGATTCGCTCCAACGAACTGGCTGACAGTGCATCCACTAAAGTTACCGAGGCTGCCCGTGTGGCAAACCAAGCTCAACAGCTTTCCAAATTCTTTGAGTAATCAAACAGGAGAAACCATTATGat                                                                                                                                                                                                                                                                                                       <  1:85524‑M1/300‑3 (MQ=255)`  
            `agaAGTTTCGCTGGAGAGTCCCATTCTAATACGACTCACTAAAGGAGACACACCATGTTAAAACTGATTAAGAAGTTAGGCCAACTGCTGGTTCGTATGTACAACGTGGAAGCCAAGCGACTGAACGATGAGGCTCGTAAAGAGGCCACACAGTCACGCGATCTGGCGATTCGCTCCAACGAACTGGCTGACAGTGCATCCACTAAAGTTACCGAGGCTGCCCGTGTGGCAAACCAAGCTCAACAGCTTTCCAAATTCTTTGAGTAATCAAACAGGAGAAACCATTATGatcgtttctgac                                                                                                                                                                                                                                                                                             <  1:427305‑M1/301‑13 (MQ=255)`  
                `gTTTCGCTGGCGAGTCCCATTCTAATACGACTCAATAAAGTAGACACACCATGTTCAAACTGATTAAGAAGTTAGGCCAACTGCTGGTTCGTATGTACAACGTGGAACCCAAGCGACTGAACGATGAGGCTCGTAAAGAGGCCACACAGTCACGCGATCTGCAGATTCGCTCCAACGAACGAGCAGAAAGTGACTCCTCTAAAGTTACAGAGGCCACACGGGTGGCAACACAAGCTCAAAAGCTCTCCCAATTCTTTGAGTCATCCACCAGGAGAACCCCTTCTgcacgctgtctcatata                                                                                                                                                                                                                                                                                         >  1:498389/1‑285 (MQ=255)`  
                 `tttCGCTGGAGAGTCCCATTCTAATACGACTCACTAAAGGAGACACACCATGTTCAAACTGATTAAGAAGTTAGGCCAACTGCTGGTTCGTATGTACAACGTGGAAGCCAAGCGACTGAACGATGAGGCTCGTAAAGAGGCCACACAGTCACGCGCTCTGGCGATTCGCTCCAACGAACTGGCTGACAGTGCATCCACTAAAGTTACCGAGGCTGCCCGTGTGGCAAACCAAGCTCACCAGCTTTCCAAATTCTTTGAGTAATCAAACAGGAGAAACCATTATGatcgtttctgacatcga                                                                                                                                                                                                                                                                                        >  1:160765‑M1/1‑284 (MQ=255)`  
                   `tCGCTGGAGAGTCCCATTCTAATACGACTCACTAAAGGAGACACACCATGTTCAAACTGATTAAGAAGTTAGGCCAACTGCTGGTTCGTATGTACAACGTGGAAGCCAAGCGACTGAACGATGAGGCTCGTAAAGAGGCTACACAGTCACGCGCTCTGGCGATTCGCTCCAACGAACTGGCTGACAGTGCATCCACTAAAGTTACCGAGGCTGCCCGTGTGGCAAACCAAGCTCAACAGCTTTCCAAATTCTTTGAGTAATCAAACAGTAGAAACCATTATGatcgtttctgacatcgaag                                                                                                                                                                                                                                                                                      >  1:133414‑M1/1‑282 (MQ=255)`  
                   `tCGCTGGAGAGTCCCATTCTAATACGACTCACTAAAGGAGACACACCATGTTCAAACTGATTAAGAAGTTAGGCCAACTGCTGGTTCGTATGTACAACGTGGAAGCCAAGCGACTGAACGATGAGGCTCGTAAAGAGGCCACACAGTCACGCGCTCTGGCGATTCGCTCCAACGAACTGGCTGACAGTGCATCCACTAAAGTTACCGAGGCTGCCCGTGTGGCAAACCAAGCTCAACAGCTTTCCAAATTCTTTGAGTAATCAAACAGGAGAAACCATTATGatcgtttctgacatcgaag                                                                                                                                                                                                                                                                                      >  1:126417‑M1/1‑282 (MQ=255)`  
                           `gagTCCCATTCTAATACGACTCACTAAAGGAGACACACCATGTTCAAACTGATTAAGAAGTTAGGCCAACTGCTGGTTCGTATGTACAACGTGGAAGCCAAGCGACTGAACGATGAGGCTCGTAAAGAGGCCACACAGTCACGCGCTCTGGCGATTCGCTCCAACGAACTGGCTGACAGTGCATCCACTAAAGTTACCGAGGCTGCCCGTGTGGCAAACCAAGCTCAACAGCTTTTCAAATTCTTTGAGTAATCAAACAGGAGAAACAATTATGatcgtttctgacatcgaagccctgtct                                                                                                                                                                                                                                                                              >  1:319843‑M1/1‑274 (MQ=255)`  
                               `cccATTCTAATACGACTCACTAAAGGAGACACACCATGTTCAAACTGATTAAGAAGTTAGGCCAACTGCTGGTTCGTATGTACAACGTGGAAGCCAAGCGACTGAACGATGAGGCTCGTAAAGAGGCCACACAGTCACGCGCTCTGGCGATTCGCTCCAACGAACTGGCTGACAGTGCATCCACTAAAGTTACCGAGGCTGCCCGTGTGGCAAACCAAGCTCAACAGCTTTCCAAATTCTTTGAGTAATCAAACAGGAGAAACCATTATGatcgtttctgacatcgaagataacgccctct                                                                                                                                                                                                                                                                          >  1:381334‑M1/1‑270 (MQ=255)`  
                                  `aTTCTAATACGACTCACTAAAGGAGACACACCATGTTCAAACTGATTAAGAAGTTAGGCCAACTGCTGGTTCGTATGTACAACGTGGAAGCCAAGCGACTGAACGATGAGGCTCGTAAAGAGGCCACACAGTCACGCGCTCTGGCGATTCGCTCCAACGAACTGGCTGACAGTGCATCCACTAAAGTTACCGAGGCTGCCCGTGTGGCAAACCAAGCTAAACAGCTTTCCAAATTCTTTGAGTCATCAAACAGGAGAAATCAATATGatcgtttctgacatcgaagctaacgccctcttag                                                                                                                                                                                                                                                                       >  1:121515‑M1/1‑267 (MQ=255)`  
                                   `ttCTAATACGACTCACTTAAGGAGACACACCATGTTCAAACTGATTAAGAATTTAGGCCAACTGCTGACTCGTATGTACAACATGGAAGCCAATCTACTGAACGATGAGGCTCGTAAAGAGGCCACACATACACGCGCTCTGGCGATTCGCTCCAACGAACTGGCTATAAGTGCATACACTAAAGTAACCGAGGCTGCCCGTGTGGCAAACCAAGCTCAACTGCTTTCCAAATTCATTGAGTACTCAAACATGAGAAATCAATATGatcgtttctcacctcatctctaaacccatcttaga                                                                                                                                                                                                                                                                      >  1:204157‑M1/1‑266 (MQ=255)`  
                                   `ttCTAATACGACTCACTAAAGGAGACACACCATGTTCAAACTGATTAAGAAGTTAGGCCAACTGCTGGTTCGTATGTACAACGTGGAAGCCAAGCGACTGAACGATGAGGCTCGTAAAGAGGCCACACAGTCACGCGCTCTGGCGATTCGCTCCAACGAACTGGCTGACAGTGCATCCACTAAAGTTACCGAGGCTGCCCGTGTGGCAAACCAAGCTCAACAGCTTTCCAAATTCTTTGAGTAATCAAACAGGCGAAATCAATATGatcgtttcttacatccaagctaacgccctcttaga                                                                                                                                                                                                                                                                      >  1:29262‑M1/1‑266 (MQ=255)`  
                                   `ttCTAATACGACTCACTAAAGGAGACACACCATGTTCAAACTGATTAAGAAGTTAGGCCAACTGCTGGTTCGTATGTACAACGTGGAAGCCAAGCGACTGAACGATGAGGCTCGTAAAGAGGCCACACAGTCACGCGCTCTGGCGATTCGCTCCAACGAACTGGCTGACAGTGCATCCACTAAAGTTACCGAGGCTGCCCGTGTGGCAAACCAAGCTCAACAGCTTTCCAAATTCTTTGAGTAATCAAACAGGAGAAACCATTATGatcgtttctgacatagaagctaacgccctattaga                                                                                                                                                                                                                                                                      >  1:368192‑M1/1‑266 (MQ=255)`  
                                   `ttCTAATACGACTCACTAAAGGAGACACACCATGTTCAAACTGATTAAGAAGTTAGGCCAACTGCTGGTTCGTATGTACAACGTGGAAGCCAAGCGACTGAACGATGAGGCTCGTAAAGAGGCCACACAGTCACGCGCTCTGGCGATTCGCTCCAACGAACTGGCTGACAGTGCATCCACTAAAGTTACCGAGGCTGCCCGTGTGGCAAACCAAGCTCAACAGCTTTCCAAATTCTTTGAGTAATCAAACAGGAGAAACCATTATGatcgtttctgacactgtctcttatacatatctccg                                                                                                                                                                                                                                                                      >  1:372205‑M1/1‑266 (MQ=255)`  
                                    `tttaaatctactcacgaacGCATACAAACCATGTGCAAACTGATTAAGAAGTTAGGCCAACTGCTGGTTCGTATGTACAACGAGGAAGCCAAGCCACTGAACGATGAGGCTCGTAAAGAGGCCACACAGTCACGCGCTCTGGCGATTCGCTCCAACGAACTGGCTGACAGTGCATCCACTAAAGTTACCGAGGCTGCCCGTGTGGCAAACCAAGCTCTACAGCTTTCCAAATTCTTTGAGTAATCAAACAGGAGAAACCATTATGatcgtttctgacatcgaagctaacgccctcttagag                                                                                                                                                                                                                                                                     <  1:353729‑M1/292‑37 (MQ=255)`  
                                    `tataaTACGACTCACTAAAGGAGAAACACCAGGTTCAAACTGATTAAGAAGTTAGGCCAACTGCTGGTTCGTATGTACAACGTGGAAGCCAAGCGACTGAAAGCTGAGGCTAGTAAAGAGGCCACACAGTCACGCGCTCTGGCGATTCGATCCAACGAACTGGCTGACAGTGCATCCACTAAAGTTACCGAGGCTGGCCGTGTGGCAAACCAAGCTCAACAGCTTTAAAAATTCTTGGAGTAATCAAACAGGAGAAACCATTATGatcgtttctgacatcgaagctaacgccctcttagag                                                                                                                                                                                                                                                                     <  1:56935‑M1/299‑37 (MQ=255)`  
                                    `tCTAATACGACTCACTAAAGGAGACACACCATGTTCAAACTGATTAAGAAGTTAGGCCAACTGCTGGTTCGTATGTACAACGTGGAAGCCAAGCGACTGAACGATGAGTCTCGTAAAGAGGCCACACAGTCACGCGCTCTGGCGATTCGCTCCAACGAACTGGCTGACAGTGCATCCACTAAAGTTACCGAGGCTGCCCGTGTGGCAAACCAAGCTCAACAGCTTTCCAAATTCTTTGAGTAATCAAACAGGAGAAATCAATATGatcgtttctgacatcgaagctaacgccctcttagag                                                                                                                                                                                                                                                                     <  1:389986‑M1/301‑37 (MQ=255)`  
                                    `tCTAATACGACTCACTAAAGGAGACACACCATGTTCAAACTGATTAAGAAGTTAGGCCAACTGCTGGTTCGTATGTACAACGTGGAAGCCAAGCGACTGAACGATGAGGCTCGTAAAGAGGCCACACAGTAACGCGCTCTGGCGATTCGCTCCAACGAACTGGCTGACAGTGCATCCACTAAAGTTACCGAGGCTGCCCGTGTGGCAAACCAAGCTCAACAGCTTTCCAAATTCTTTGAGTAATCAAACAGGAGAAACCATTATGatcgtttctgacatcgaagctaacgccctcttagag                                                                                                                                                                                                                                                                     <  1:336531‑M1/301‑37 (MQ=255)`  
                                    `tCTAATACGACTCACTAAAGGAGACACACCATGTTCAAACTGAGTAAGAAGTGAGGCCAACTGCGGGGTCGTATGTAAAACGGGGAAGCCAAGCGACTGAACGATGAGGCGCGTAAAGAGGCCACACAGTCACGCGCTCTGGCGATTCGATCCAACGAACTGGCTGACAGTGCATCCACTAAAGTTACCGAGGCTGCCCGTGTGGCAAACCAAGCTCAACAGCTTTCCAAATTCTTTGAGTAATCAAACAGGAGAAATCAATATGatcgtttctgacatcgaagctaacgccctcttagag                                                                                                                                                                                                                                                                     <  1:452857‑M1/301‑37 (MQ=255)`  
                                                `cACTAAAGGAGACACACCATGTTCAAACTGATTAAGAAGTTAGGCCAACTGCTGGTTCGTATGTACAACGTGGAAGCCAAGCTACTGAACGATGAGGCTCGTAAAGAGGCCACACAGTCACGCGCTCTGGCGATTCGCTCCAACGAACTGGCTGACAGTGCATCCACTAAAGTTACCGAGGCTGCCCGTGTGGCAAACCAAGCTCAACAGCTTTCCAAATTCTTTGAGTAATCCAACAGGAGAAATCAATATGatcgtttctaacatcaacgctaactccctcttagagaccgtcactaat                                                                                                                                                                                                                                                         >  1:331776‑M1/1‑253 (MQ=255)`  
                                                `cACTAAAGGAGACACACCATGTTCAAACTGATTAAGAAGTTAGGCCAACTGCTGGTTCGTATGTACAACGTGGAAGCCAAGCGACTGAACGATGAGGCTCGTAAAGAGGCCACACAGTCACGCGCTCTGGCGATTCGCTCCAACGAACTGGCTGACAGTGCATCCACTAAAGTTACCGAGGCTGCCCGTGTGGCAAACCAAGCTCAACAGCTTTCCAAATTCTTTGAGTAATCAAACAGGAGAAATCAATATGatcgtttctgacatcgaagctaacgccctcttagagctgtctcttata                                                                                                                                                                                                                                                         >  1:214275‑M1/1‑253 (MQ=255)`  
                                                 `aCTAAAGGAGACACACCATGTTCAAACTGATTAAGAAGTTAGGCCAACTGCTGGTTCGTATGTACAACGTGGAAGCCAAGCGACTGAACGATGAGGCTCGTAAAGAGGCCACACAGTCACGCGCTCTGGCGATTCGCTCCAACGAACTGGCTGACAGTGCATCCACTAAAGTTACAGAGGCTGCCCGTGTGGCAAACCAAGCTCAACAGCTTTCCAAATTCTTTGAGTAATCAAACAGGAGAAACCATTATGatcgtttctgacatcgaagctaacgccctcttagagagcgtcactaaat                                                                                                                                                                                                                                                        >  1:143158‑M1/1‑252 (MQ=255)`  
                                                       `tcggagatgtgtataagagacagattaagaagttaggccaactgcTGGTTCGTATGTACAACGTGGAAGCCAAGCGACTGAACGATGAGGCTCGTAAAGAGGCCACACAGTCACGCGCTCTGGCGATTCGCTCCAACGAACTGGCTGACAGTGCATCCACTAAAGTTACCGAGTCTGCACGTGT‑GCAAACCAAGCTCAACAGCTTTCCAAATTCTTTGAGTAATCAAACAGGAGAAATCAATATGatcgtttctgacatcgaagctaacgacctcttagagagcgtcactaagttccactg                                                                                                                                                                                                                                                 <  1:11327‑M1/279‑57 (MQ=255)`  
                                                         `agaaataacatgttaaaAGTGATTAAGAGGTTAGGACAAGCGCTGGATCGTATGTACAACGTGGAAGGCAAGCGAGTGAACGATGGGGCTCGTAAAGAAGCCACAAGGTAACGCGCTCTGGCGATTCGGTCCGACGAACTGGCTGACAGTGCATCCACTAAAGTTACCGAGTATGACCGTGTGACAATGCAAGCTAAACAGCTTTCCAAATTCTTTGAGAAATCAAACAGGAGAAACCAGTATGatcgtttctgacatcgaagctaacgccctcttagagagcgtcactaagttccactgc                                                                                                                                                                                                                                                <  1:305933‑M1/293‑58 (MQ=255)`  
                                                          `gaCACACCATGTTCAAACTGATTAAGAAGTTAGGCCAACTGCTGGTTCGTATGTACAACGTGGAAGCCAAGCGACTGAACGATGAGGCTCGTAAAGAGGCCACACAGTCACGCGCTCTGGCGATTCGCTCCAACGAACTGGCTGACAGTGCATCCACTAAAGTTACCGAGGCTGCCCGTGTGGCAAACCAAGCTCAACAGCTTTCCAAATTCTTTGAGTAATCAAACAGGAGAAACCATTATGatcgtttctgacatcgaagcttacgccctcttagagagcgtcactaagttccactccg                                                                                                                                                                                                                                               >  1:431841‑M1/1‑243 (MQ=255)`  
                                                           `acacacCATGTTCAAACTGATTAAGAAGTTAGGCCAACTGCTGGTTCGTATGTACAACGTGGAAGCCAAGCGACTGAACGATGAGGCTCGTAAAGAGGCCACACAGTCACGCGCTCTGGCGATTCGCTCCAACGAACTGGCTGACAGTGCATCCACTAAAGTTACCGAGGCTGCCCGTGTGGCAAACAAAGCTCAACAGCTTTCCAAATTCTTTGAGTAATCAAACAGGAGAAACCATTATGatcgtttctgacatcgaagctaacgccctcttagagagcgtcactaagttccactgcgg                                                                                                                                                                                                                                              >  1:413094‑M1/1‑242 (MQ=255)`  
                                                                `ccATGTTCAAACTGATTAAGAAGTTAGGCCAACTGCTGGTTCGTATGTACAACGTGGAAGCCAAGCGACTGAACGATGAGGCTCGTAAAGAGGCCACACAGTCACGCGCTCTGGCGATTCGCTCCAACGAACTGGCTGACAGTGCATCCACTAAAGTTACCGAGGCTGCCCGTGTGGCAAACCAAGCTCAACAGCTTTCCAAATTCTTTGAGTAATCAAACAGGAGAAACCATTATGatcgtttctgacatcgaagctaacgccctcttagagagcgtcactaagttccactgcggggtta                                                                                                                                                                                                                                         >  1:443865‑M1/1‑237 (MQ=255)`  
                                                                   `tGTTCAAACTGATTAAGAAGTTAGGCCAACTGCTGGTTCGTATGTACAACGTGGAAGCCAAGCGACTGAACGATGAGGCTCGTAAAGAGGCCACACAGTCACGCGCTCTGGCGATTCGCTCCAACGAACTGGCTGACAGTGCATCCACTAAAGTTACCGAGGCTGCCCGTGTGGCAAACCAAGCTCAACAGCTTTCCAAATTCTTTGAGTAATCAAACAGGAGAAACCATTATGatcgtttctgacatcgaagctaacgccctcttagagagcgtcactaagttccactgcggggttatct                                                                                                                                                                                                                                      >  1:146565‑M1/1‑234 (MQ=255)`  
                                                                   `tGTTCAAACTGATTAAGAAGTTAGGCCAACTGCTGGTTCGTATGTACAACGTGGAAGCCAAGCGACTGAACGATGAGGCTCGTAAAGAGGCCACACAGTCACGCGCTCTGGCGATTCGCTCCAACGAACTGGCTGACAATGCATCCACTAAAGTTACCGAGGCTGCCCGTGTGGCAAACCAAGCTCAACAGCTTTCCAAATTCTTTGAGTAATCAAACAGGAGAAACCATTATGatcgtttctgacttcgaagctcacgccatcttagtgagcgtcactaagttccactgcggggtctgtt                                                                                                                                                                                                                                      >  1:346441‑M1/1‑234 (MQ=255)`  
                                                                   `tGTTCAAACTGATTAAGAAGTTAGGCCAACTGCTGGTTCGTATGTACAACGTGGAAGCCAAGCGACTGAACGATGAGGCTCGTAAAGAGGCCACACAGTCACGCGCCCTGGCGATTCGCTCCAACGAACTGGCTCACAGTGCATCCACTAAAGTTACCGAGGCTGACCGTGTGGCAAACCACGCTCAACAGCTTTACAACTCCTTTGAGTTCTCAAACAGTAGAAATCAATATGatcgtttctgacatcgtagataactccctcttagagagcgtcactaagttccactgcgggtttatct                                                                                                                                                                                                                                      >  1:296237‑M1/1‑234 (MQ=255)`  
                                                                    `gTTCAAACTGATTAAGAAGTTAGGCCAACTGCTGGTTCGTATGTACAACGTGGAAGCCAAGCGACTGAACGATGAGGCTCGTAAAGAGGCCACACAGTCACGCGCTCTGGCGATTCGCTCCAACGAACTGGCTGACAGTGCATCCACTAAAGTTACCGAGGCTGCCCGTGTGGCAAACCAAGCTCAACAGCTTTCCAAATTCTTTGAGTAATCAAACCGGAGAAACCATTATGatcgtttctgacatcgaagctaacgccctcttagagagcgtcactaagttccactgcggggtctgtct                                                                                                                                                                                                                                     >  1:109691‑M1/1‑233 (MQ=255)`  
                                                                        `aaaCTGATTAAGAAGTTAGGCCAACTGCTGGTTCGTATGTACAACGTGGAAGCCAAGCGACTGAACGATGAGGCTCGTAAAGAGGCCACACAGTCACGCGCTCTGGCGATTCGCTCCAACGAACTGGCTGACAGTGCATCCACTAAAGTTACCGAGGCTGCCCGTGTGGCAAACCAAGCTCAACAGCTTTCCAAATTCTTTGAGTAATCAAACAGGAGAAACCATTATGatcgtttctgacatcgaagctaacgccctcttagagagcgtcactaagttccactgcggggttatctacgac                                                                                                                                                                                                                                 <  1:440104‑M1/301‑73 (MQ=255)`  
                                                                        `aaaCTGATTAAGAAGTTAGGCCAACTGCTGGTTCGTATGTACAACGTGGAAGCCAAGCGACTGAACGATGAGGCTCGTAAAGAGGCCACACAGTCACGCGCTCTGGCGATTCGATCCAACGAACTGGCTGACAGTGCATCCACTAAAGTTACCGAGGCTGCCCGTGTGGCAAACCAAGCTCAACAGCTTTCCAAATTCTTTGAGTAATCAAACAGGAGAAACCATTATGatcgtttctgacatcgaagctaacgccctcttagagagcgtcactaagttccactgcggggttatctacgac                                                                                                                                                                                                                                 <  1:350809‑M1/301‑73 (MQ=255)`  
                                                                           `cTGATTAAGAAGTTAGGCCAACTGCTGGTTCGTATGTACAACGTGGAAGCCAAGCGACTGAACGATGAGGCTCGTAAAGAGGCCACACAGTCACGCGCTCTGGCGATTCGCTCCAACGAACTGGCTGACAGTGCATCCACTAATGTTACCGAAGCTGCCCGTGTGGCAAACCACGCTCAACAGCTTTCCAAATTCTTTGAGTAATCAAACAGGAGAAACAATTATGatcgtatcttacatcgaagctaacgccctcttagatagcgcccctaagttcccctgcggggttatctacgactac                                                                                                                                                                                                                              >  1:80947‑M1/1‑226 (MQ=255)`  
                                                                             `gATTAAGAAGTTAGGCCAACTGCTGGTTCGTATGTACAACGTGGAAGCCAAGCGACTGAACGATGAGGCTCGTAAAGAGGCCACACAGTCACGCGCTCTGGCGATTCGCTCCAACGAACTGGCTGACAGTGCATCCACTAAAGTTACCGAGGCTGCCCGTGTGGCAAACCAAGCTCAACAGCTTTCCAAATTCTTTGAGTAATCAAACAGGAGAAACCATTATGatcgtttctgacatcgaagctaacgccctcttagagagcgtcactaagttccactgcggggttatctacgactactc                                                                                                                                                                                                                            >  1:151971‑M1/1‑224 (MQ=255)`  
                                                                                    `aagTTAGGCCAACTGCTGGTTCGTATGTACAACGTGGAAGCCAAGCGACTGAACGATGAGGCTCGTAAAGAGGCCACACAGTCACGCGCTCTGGCGATTCGCTCCAACGAACTGGCTGACAGTGCATCCACTAAAGTTACCGAGGCTGCCCGTGGGGCAAACCAAGCTCAACAGCTTTCCAAATTCTTTGAGTAATCAAACAGGAGAAACCATTATGatcgtttctgacatcgaagctaacgccctcttagagagcgtcactaagttccactgcggggttatctacgactctgtctcttat                                                                                                                                                                                                                     >  1:272067‑M1/1‑217 (MQ=255)`  
                                                                                     `agTTAGGCCAACTGCTGGTTCGTATGTACAACGTGGAAGTCAAGCGACTGAACGATGAGGCTCGTAAAGAGGCCACACAGTCACGCGCTCTGGCGATTCGCTCCAAAGAACTGGCTGACAGTGCATCCACTAAAGTTACCGAGGCTGCCCGTGTGGCAAACCAAGCTCAACAGCTTTCCAAATTCTTTGAGTAATCAAACAGGAGAAACCATTATGatcgtttctgacatcgaagctaacgccctcttagagagcgtcactaagttccactgcggggttatctacgactactccaccgctg                                                                                                                                                                                                                    <  1:464922‑M1/301‑86 (MQ=255)`  
                                                                                             `tataagagacagcggatgtacaacgttgaagCCAAGCGACTGAACGATGAGGCTCGTAAAGAGGCCACACAGTCACGCGCTCTGGCGATTCGCTGCAACGAAATGGCTGACAGTGCATCCACTAAAGTTACCGAGGCTGCCCGTGTGGCAAACCAAGCTCAACAGCTTTCCAAATTCTTTGAGTAATCAAACAGGAGAAACCATTATGagcgtttctgacatcgaagctaacgccctcttagagagcgtcactaagttccactgcggggttatctacgactactccaccgctgagtacgta                                                                                                                                                                                                            <  1:174336‑M1/286‑94 (MQ=255)`  
                                                                                               `aCTGCTGGTTCGTATGTACAACGTGGAAGCCAAGCGACTGAACGATGAGGCTCGTAAAGAGGCCACACAGTCACGCGCTCTGGCGATTCGCTCCAACGAACTGGCTGACAGTGCATCCACTAAAGTTACCGAGGCTGCCCGTGTGGCAAACCAAGCTCAACAGCTTTCCAAATTCTTTGAGTAATCAAACAGGAGAAATCAATATGatcgtttctgacatcgaagctaacgccctcttagagagcgtcactaagttccactgcggggttatctacgactactccaccgctgagtacgtaag                                                                                                                                                                                                          <  1:456696‑M1/301‑96 (MQ=255)`  
                                                                                                `ctgctgGTTCGTATGTACAACGTGGAAGCCAAGCGACTGAACGATGAGGCTCGTAAAGAGGCCACACAGTCACGCGCTCTGGCGATTCGCTCCAACGAACTGGCTGACAGTGCATCCACTAAAGTTACCGAGGCTGCCCGTGTGGCAAACCAAGCTCAACAGCTTTCCAAATTCTTTGAGTAATCAAACAGGAGAAACCATTATGatcgtttctgacatcgaagctaacgccctcttagagagcgtcactaagttccactgcggggttatctacgactactccactgctgagtacgtaagc                                                                                                                                                                                                         >  1:61706‑M1/1‑205 (MQ=255)`  
                                                                                                `ctgctgGTTCGTATGTACAACGTGGAAGCCAAGCGACTGAACGATGAGGCTCGTAAAGAGGCCACACAGTCACGCGCTCTGGCGATTCGCTCCAACGAACTGGCTGACAGTGCATCCACTAAAGTTACCGAGGCTGCCCGTGTGGCAAACCAAGCTCAACAGCTTTCCAAATTCTTTGAGTAATCAAACAGGAGAAACCATTATGatcgtttctgacatcgaagctaacgccctcttagagagcgtcactaagttccactgcggggttatctacgactactccacccgtctcttatacaca                                                                                                                                                                                                         >  1:473433‑M1/1‑205 (MQ=255)`  
                                                                                                         `cGTATGTACAACGTGGAAGCCAATCGACTGAACGATGAGGCTCGTAAAGAGGCCACACAGTCACGCGCTCTGGCGATTCGCTCCAACGAACTGGCTGACAGTGCATCCACTAAAGTTACCGAGGCTGCCCGTGTGGCAAACCAAGCTCAACAGCTTTCCAAATTCTTTGAGTAATCAAACAGGAGAAACCATTATGatcgtttctgacatcgaagctaacgccctcttagagagcgtcactaagttccactgcggggttatctacgactactccaccgctgagtacgtaagctaccgtccg                                                                                                                                                                                                >  1:479294‑M1/1‑196 (MQ=255)`  
                                                                                                         `cGTATGTACAACGTGGAAGCCAAGCGACTGAACGATGAGGCTCGTAAAGAGGCCACACAGTCACGCGCTCTGGCGATTCTCTCCAACGAACTGGCTGACAGTGCATCCACTAAAGTTACCGAGGCTGCCCGTGTGGCAAACCAAGCTCAACAGCTTTCCAAATTCTTTGAGTAATCAAACAGGCGAAACCATTATGatcgtttctgacatcgaagctaacgccctcttagagagcgtcactaagttccacggcggggttatatacgactactccaccgctgagtacgtaagctaacgtccg                                                                                                                                                                                                >  1:253539‑M1/1‑196 (MQ=255)`  
                                                                                                         `cGTATGTACAACGTGGAAGCCAAGCGACTGAACGATGAGGCTCGTAAAGAGGCCACACAGTCACGCGCTCTGGCGATTCGCTCCAACGAACTGGCTGACAGTGCAGCCACTAAAGTGACCGAGGCTGCCCGTGTGGCAAACCAAGCTCAACAGCTTTCAAAATTCTTTGAGTAATCAAACAGGAGAAAGCAATATGatcgtttctgacatcgaagctaacgccctcttagagagcgtcactaagttccacttcggggttatctacgactactacaccgatgaggacgtacgctaacgtccg                                                                                                                                                                                                >  1:408454‑M1/1‑196 (MQ=255)`  
                                                                                                           `taggtacAACGTGGAAGCCAAGCGACTGAACGATAAGGCTCGTAAAGATGCCACACAGTCACGCGCTCTGGCGATTCGCTCCAACGAACTGGCTGAACGTGCATCCACTAAACTTACCGAGGCTGCCCGTGTGGCAAACCAAGCTCAACAGCTTTCCAAATTCTTTGAGCAATCAAACAGGAGAAACCATGATGatcgtttctgacatcgaagctaacgccctcttagagatcgtcactaagttccactgcggggttatctacgactactccaccgctgagtacgtaagctaccgtccgag                                                                                                                                                                                              <  1:156532‑M1/298‑108 (MQ=255)`  
                                                                                                           `tATTTACAACCTGGAAGCCAAGCGACTGAACGATGAGGCTCGTAAAGAGGCCACACAGTCACGCGCTCTGGCGAGTCGCTCCAACGAACTGGCTGACAGTGCATCCACTAAAGTTACCGAGGCTGCCCGTGTGGCAAACCAAGCTCAACAGCTTTCCAAATTCTTTGAGTAATCAAACAGGAGAAATCAATATGatcgtttctgacatcgaagctaacgccctcttagagagcgtcactaagttccactgcggggttatctacgactactcaacagctgagtacgtaagctaccgtccgag                                                                                                                                                                                              <  1:19445‑M1/301‑108 (MQ=255)`  
                                                                                                             `tGTACAACGTGGAAGCCAAGCGACTGAACGATGAGGCTCGTAAAGAGGCCACACAGTCACGCGCTCTGGCGATTCGCTCCAACGAACTGGCTGACAGTGCATCCACTAAAGTTACCGAGGCTGCCCGTGTGGCAAACCAAGCTCAACAGCTTTCCAAATTCTTTGAGTAATCAAACAGGAGAAACCATTATGatcgtttctgacatcgaagctaacgccctcttagagagcgtcactaagttccactgcggggttatctacgactactccaccgctgagtacgtaagctacctgtctctta                                                                                                                                                                                            >  1:263304‑M1/1‑192 (MQ=255)`  
                                                                                                              `gTACAACGTGGAAGCCAAGCGACTGAACGATGAGGCTCGTAAAGAGGCCACACAGTCACGCGCTCTGGCGATTCGCTCCAACGAACTGGCTGACAGTGTATCCACTAAAGTTACCGAGGCTGCCCGTGTGGCAAACCAAGCTCAACAGCTTTCCAAATTCTTTGAGTAATCAAACAGGAGAAACCATTATGatcgtttctgacatcgaagctaacgccctcttagagagcgtcactaagttccactgcggggttatctacgactactccaccgctgagtacgtaagctaccgtccgagtga                                                                                                                                                                                           >  1:259648‑M1/1‑191 (MQ=255)`  
                                                                                                                  `aaCGAGGAAGCCAAGCGACTGAACGATGAGGCTCGTAAAGAGGCCACACAGTAACGCGCTCTGGCGATTCGCTCCAACGAACTGGCTGACAGTGCATCCACTAAAGTTACAGAGGCTGCCCGTGTGGCAAACCAAGCTCAACAGCTTTCCAAATTCTTTGAGTAATCAAACAGGAGAAACAATTATGatcgtttctgacatcgaagctaacgccctcttagagagcgtcactaagttccactgcggggttatctacgactactccaccgctgagtacgtaagctaccgtccgagtgacttc                                                                                                                                                                                       <  1:508719‑M1/301‑115 (MQ=255)`  
                                                                                                                            `ccAAGCGACTGAACGATGAGGCTCGTAAAGAGGCCACACAGTCACGCGCTCTGGCGATTCGCTCCAACGAACTGGCTGACAGTGCATCCACTAAAGTTACCGAGGCTGCCCGTGTGGCAAACCAAGCTCAACAGCTTTCCAAATTCTTTGAGTAATCAAACAGGAGAAACCATTATGatcgtttctgacatcgaagctaacgccctcttagagagcgtcactaagttccactgcggggttatctacgactactccaccgctgagtacgtaagctaccgtccgagtgacttcggtgcgtatc                                                                                                                                                                             >  1:139394‑M1/1‑177 (MQ=255)`  
                                                                                                                                `gCGACTGAACGATGAGGCTCGTAAAGAGGCCACACAGTCACTCGCTCTGGCGATTCGCTCCAACGAACTGGCTGACAGTGCAGCCACTAAAGTTACCGAGGCTGCCCGTGTGGCAAACCAAGCTCAACAGCTTTCCAAATTCTTTGAGTAATCAAACAGGAGAAACCATTATGatcgtttctgacatcgaagctaacgccctcttagagagcgtcactaagttccactgcggggttatctacgactactccaccgctgagtacgtaagctaccgtccgagtgacttcggtgcgtatctgga                                                                                                                                                                         <  1:418695‑M1/301‑129 (MQ=255)`  
                                                                                                                                `gCGACTGAACGAGGAGGCTCGTAAAGAGGCCACACAGTCACGCGCTCGGGCGATTCGCTCCAACGAACTAGCTGACAGTGCATCCACTAAAGTTACCGAGGCTGCCCGTGTGGCAAACCAAGATCAACAGCTTTCCAAATTCTTTGAGTAATCAAACAGGAGAAACCATTATGatcgtttctgacatcgaagctaacgccctcttagagagcgtcactaagttccactgcggggttatctacgactactccaccgctgagtacgtaagctaccgtccgagtgacttcggtgcgtatctgga                                                                                                                                                                         <  1:243821‑M1/301‑129 (MQ=255)`  
                                                                                                                                 `cGACTGAACGATGAGGCTCGTAAAGAGGCCACACAGTCACGCGCTCTGGCGATTCGCTCCAACGAACTGGCTGACAGTGCATCCACTAAAGTTACCGAGGCTGCCCGTGTGGCAAACCAAGCTCCACAGCTTTCCAAGTTCTTTTAGTAATCAAACAGGAGAAACCATTATTatcttttctgacatcgaagctaacgccctcttagatagcgtcactaagttccactgcggggttatctacgactactccaccgctgagtacgtaagctaccgtccgagtgacttcggctgtctcttatac                                                                                                                                                                        >  1:230577‑M1/1‑172 (MQ=255)`  
                                                                                                                                 `cGACTAAACGAGGAGCCTCGTAAGGAGGCCACACCGTCAGGCGCTCTGGCGATTCGCTCCAACGAACTGGCTGACAGTTCATCCACTAAAGTTACCGAGGCTGCCCGTGTGGCAAACCAAGCTCAACAGCTTTCCAAGTTCTTTGAGTAATCAAACAGGAGAAACCATTATGatcgtttctgacatcgaagctaacgccctcttagagagcgtcactaagttccactgcggggttatctacgactactccaccgctgagtacgtaagctaccgtccgagtgacttcggtgcgtatctggat                                                                                                                                                                        <  1:398724‑M1/301‑130 (MQ=255)`  
                                                                                                                                          `gagacagctcgtaAAGAGGCCACACAGTCACGCGCTCTGGCGATTCGCTCCAACGAACTGGCTGACAGTGCATCCACTAAAGTTACCGAGGCTGCCCGTGTGGCAAACCAAGCTCAACAGCTTTCCAAATTCTTTGAGTAATCAAACAGGAGAAACCATTATGatcgtctctgacatcgaagctaacgccctcttagagagcgtcactaagttccactgcggggttatctacgactactccaccgctgagtacgtaagctaccgtccgagtgacttcggtgcgtatctggatgcgctggaa                                                                                                                                                               <  1:277580‑M1/295‑139 (MQ=255)`  
                                                                                                                                                 `cTCGTAAAGAGGCCACACAGTCACGCGCTCTGGCGATTCGCTCCAACGAACTGGCTGACAGTGCATCCACTAAAGTTACCGAGGCTGCCCGTGTGGAAAACCAAGCTCAACAGCTTTCCCAATTCTTTGAGTAATCAAACAGGAGAAACCATTATGatcgtttctgacatcgaagctaacgccctcttagagagcgtcactaagttccactgcggggttatctacgactactccaccgctgagtacgtaagctaccgtccgagtgacttcggtgcgtatcctgtctcttctacacatctcc                                                                                                                                                        >  1:167183‑M1/1‑156 (MQ=255)`  
                                                                                                                                                         `gagGCCACACAGTCACGCGCTCTGGCGATTCGCTCCAACGAACTGGCTGACAGTGCATCCACTAAAGTTACCGAGGCTGCCCGTGTGGCAAACCAAGCTCTACAGCTTTCCAAATTCTTTGAGTAATCAAACAGGAGAAACCATTATGatcgtttctgacatcgaagctaacgccctcttaacgagcgtccctaagttccactgcggggttatctacgactactccaccgctgagtccgtaagctaccgtccgagtgacttcggtgcgtatctggatgcgctggaatccgaggttgcacga                                                                                                                                                >  1:28898‑M1/1‑148 (MQ=255)`  
                                                                                                                                                                `agcagtCACGCGCGCTGGCGATTCGCTCCAACGAACTGGCTGACAGTGCATCCACTAAAGTTACCGAGGCTGCCCGTGTGGCAAACCAAGCTCAACAGCTTTCCAAATTCTTTGAGTAATCAAACAGGAGAAATCAATATGatcgtttctgacatcgaagctaacgccctcttagagagcgtcactaagttccactgcggggttatctacgactactccaccgctgagtacgtaagctaccgtccgagtgacttcggtgcgtatctggatgcgctggaagccgaggttgcacgaggcggtc                                                                                                                                         <  1:91271‑M1/299‑161 (MQ=255)`  
                                                                                                                                                                      `cACTCGCTCTGGCGATTCGCTCCAACGAACTGGCTGACAGTGCATCCACTAAAGTTACCGAGGCTGCCCGTGTGGCAAACCAAGCTCAACAGCTTTCCAAATTCTTTGAGTAATCAAACAGGAGAAACCATTATGatcgtttctgacatcgaagctaacgccctcttagagagcgtcactaagttccactgcgtggttatctacgactactccaccgctgagtacgtaagctaccgtccgagtgacttcggtgcgtatctggatgcgctggaagccgaggttgcacgaggcggtcttattg                                                                                                                                   <  1:269026‑M1/301‑167 (MQ=255)`  
                                                                                                                                                                      `cACGCGATCTGCGGATTCGCTCCAACGAACTGGCTGACAGTTCATCCACTAAAGTTACCGAGGCGGCCCGTGTGGCAAACCAAGCTCAACAGCTTTCCAAATTCTTTGAGTAATCAAACAGGAGAAACCATTATGatcgtttctgacatcgaagctaacgccctcttagagagcgtcactaagttccactgcggggttatctacgactactccaccgctgagtacgtaagctaccgtccgagtgacttcggtgcgtatctggatgcgctggaagccgaggttgcacgaggcggtcttattg                                                                                                                                   <  1:184859‑M1/301‑167 (MQ=255)`  
                                                                                                                                                                      `cACGAGCTCTGGCGATTCGCTCCAACGAACTGGCTGACAGTGCATCCACTAAAGTTACCGAGGCTGCCCGTGTGGCAAACCAAGCTCAACAGCTTTCCAAATTCTTTGAGTAATCAAACAGGAGAAACCATTATGatcgtttctgacatagaagctaacgacctcttagagagcgtcactaagttccactgcggggttatctacgactactccaccgctgagtacgtaagctaccgtccgagtgacttcggtgcgtatctggatgcgctggaagccgaggttgcacgaggcggtcttattg                                                                                                                                   <  1:422812‑M1/301‑167 (MQ=255)`  
                                                                                                                                                                         `ccgctCTGGCGATTCGCTCCAACGAACTGGCTGACAGTGCATCCACTAAAGTTACCGAGGCTGCCCGTGTGGCAAACCAAGCTCAACAGCTTTCCAAATTCTTTGAGTAATCAAACAGGAGAAACCATTATGatcttttctgacatcgaagctaacgccctcttagagagcgtcactaagttccactgcggggttatctacgactactccaccgctgagtacgtaagctaccgtccgagtgacttcggtgcgtatctggatgcgctggaagccgaggttgcacgaggcggtcttattgtgt                                                                                                                                <  1:314164‑M1/300‑170 (MQ=255)`  
                                                                                                                                                                                `ggCGATTCGCTCCAACGAACTGGCTGACAGTGCATCCACTAAAGTTACCGAGGCTGCCCGTGTGGCAAACCAAGCTCAACAGCTTTCCAAATTCTTTGAGTAATCAAACAGGAGAAATCAATATGatcgtttctggcctcgaagctaacgccctcttagagatcgtcactaagttccactgcggggttatctacgactactccaccgctgagtacgtaagctaccgtccgagtgacatcggtgcgtatccggatgcgctggaagccgagcttgaacgaggcctgcctcttatacccatctc                                                                                                                         >  1:238056‑M1/1‑125 (MQ=255)`  
                                                                                                                                                                                `ggCGATTCGCTCCAACGAACTGGCTGACAGTGCATCCACTAAAGTTACCGAGGCTGCCCGTGTGGCAAACCAAGCTCAACAGCTTTCCAAATTCTTTGAGTAATCAAACAGGAGAAACCATTATGatcgtttctgacatcgaagctaacgccctcttagagagcgtcactaagttccactgcggggttatctacgactactccaccgctgagtacgtaagctaccgtccgagtgacttcggtgcgtatctggatgcgctggaagccgaggttgcacgaggcggtcttattgtgttccacaa                                                                                                                         <  1:46154‑M1/301‑177 (MQ=255)`  
                                                                                                                                                                                          `tCCAACGAACTGGCTGACATTGCATCCACTAAAGTTACCGAGGCTGCCCGTTTGGCAAACCAAGCTCAACATCTTTCCAAATTCTTTGAGTAATCAAACAGGAGAAACCATTATGatcgtttctgacatcgaagataacgccctcttagagagcgtcactaagttccactgcggggttatctacgactactccaccgctgagtacgtaagctaccgtccgagtgacttaggtgcgtatctggatgcgctggaagccgaggttgcacgaggcggtcttattgtgttccacaacggtcacaag                                                                                                               <  1:130284‑M1/301‑187 (MQ=255)`  
                                                                                                                                                                                           `ccAACGAACTGGCTGACAGTGCATCCACTAAAGTTACCGAGGCTGCCCGTGTGGCAAACCAAGCTCAACAGCTTTCCAAATTCTTTGAGTAATCAAACAGGAGAAACCATTATGatcgtttctgacatcgaagctaacgccctcttagagagcgtcactaagttccactgcggggttatctacgactactccaccgctgagtacgtaagctaccgtccgagtgacttcggttcgtatctggatgcgctggaagccgaggttgcacgaggcggtcttattgtgttccacaacggctttctct                                                                                                              >  1:52415‑M1/1‑114 (MQ=255)`  
                                                                                                                                                                                           `ccAACGAACTGGCTGACAGTGCATCCACTAAAGTTACCGAGGCTGCCCGTGTGGCAAACCAAGCTCAACAGCTTTCCAAATTCTTTGAGTAATCAAACAGGAGAAACCATTATGatcgtttctgacatcgaagctaacgccctcttagagagcgtcactaagttccactgcggggttatctacgactactccaccgctgagtacgtaagctaccgtccgagtgacttcggtgcgtatctggatgcgctggaagccgaggttgcacgaggcggtcttattgtgttccacaacggtcacaagt                                                                                                              <  1:39546‑M1/301‑188 (MQ=255)`  
                                                                                                                                                                                            `aagagacagggctgacagtgCATCCACTAAAGTTACCGAGGCTGCCCGTGTGGCAAACCAAGCTCAACAGCTTTCCAAATTCTTTGAGTAATCAAACAGGAGAAACCATTATGatcgtttctgacatcgaagctaacgccctcttagagagcgtcactaagttccactgcggggttatctacgactactccaccactgagtacgtaagctaccgtccgagtgacttcggtgcgtatctggatgcgctggaagccgaggttgcacgaggcggtcttattgtgttccacaacggtcacaagta                                                                                                             <  1:403900‑M1/292‑189 (MQ=255)`  
                                                                                                                                                                                               `ggctcggagatgtgtataagagacagaagttaccgaggctgcccgtgtggcaaaCCAAGCTCAACAGCTTTCCAAATTCTTTGAGTAATCAAACAGGAGAAACCATTATGatcgtttctgacatcgaagctaacgccctcttagagagcgtcactaagttccactgcggggttatctacgactactccaccgctgagtacgtaagctaccgtccgagtgacttcggtgcgtatctggatgcgctggaagccgaggttgcacgaggcggtcttattgtgttccacaacggtcacaagtatga                                                                                                          <  1:198731‑M1/275‑192 (MQ=255)`  
                                                                                                                                                                                                `ttaaaagagacaggtgcatccactaaaGTTACCGAGGCTGCCCGTGTGGCAACCCAAGCTCAACAGCTTTCCAAATTCTTTGAGTAATCAAACAAGAGAAACCATTATGatcgtttctgacatcgaagctaacgccctcttagagagcgtcactaagttccactgcggggttatctacgactactccaccgctgagtacgtaagctaccgtccgagtgacttcggtgagtatctggatgcgctggaagccgaggttgcacgaggcggtcttattgtgttccacaacggtcacaagtatgac                                                                                                         <  1:485813‑M1/288‑193 (MQ=255)`  
                                                                                                                                                                                                `aaacTGGCTGACAGTGCATCCACTAAAGTTACCGAGGCTGCCCGTGTGGCAAACCAAGCTCAACAGCTTTCCAAATACTTGGAGTAATCAAACAGGAGAAAGCAATATGctcgtttttgacatcgaagctaacgccctcttagatagcgtcactaagtcccactgcggggttatctacgactactccaccgctgagtacgtaatctaccgtccgagtgacttcgatgcgtatctggatgcgctggaagccgaggttgcacgaggcggtcttattgtgttccacaacggtcacaagtatgac                                                                                                         <  1:95322‑M1/300‑193 (MQ=255)`  
                                                                                                                                                                                                 `aaagggctgaCAGTGCATCCGCTAAAGTTACCGAGGCTGCCCGTGTGGCAAACCAAGCTCAACAGCTTTCCAAAGTCTTTGAGTAATCAAACAGGAGAAACCATTATGatcgtgtctgacatcgaagctaacgacctcttagagagcgtcactaagttccactgcggggttatctacgactactccaccgctgagtacgtaagctaccgtccgagtgacttcggtgcgtatctggatgcgctggaagccgaggttgcacgatgcggtcttattgtgttccacaacgggcacaagtatgacg                                                                                                        <  1:233684‑M1/297‑194 (MQ=255)`  
                                                                                                                                                                                                 `aaCTGGCTGACAGTGCATCCACTAAAGTTACCGAGGCTGCCCGTGTGGCAAACCAAGCTCAACAGCTTTCCAAATTCTTTGAGTAATCAAACAGGAGAAACCATTATGatcgtttctgacatcgaagctaacgccctcttagagagcttcactaagttccactgcggggttatctacgactactccaccgctgagtacgtaagctaccgtccgagtgacttcggtgcgtatctggatgcgctggaagccgaggttgcacgaggcggtcttattgtgttccataacggtcacaagtatgacg                                                                                                        <  1:50379‑M1/301‑194 (MQ=255)`  
                                                                                                                                                                                                 `aaCTGGCTGACAGTGCATCCACTAAAGTTACCGAGGCTGCCCGTGTGGCAAACCAAGCTCAACAGCTTTCCAAATGCTTTGAGTAATCAAACAGGAGAAACCATTATGatcgtttctgacatcgaagctaacgccctcttagagagcgtcactaagttccactgcggggttatctacgactactccaccgctgagtacgtaagctaccgtccgagtgacttcggtgcgtatctggatgcgctggaagccgaggttgcacgaggcggtcttattgtgttccacaacggtcacaagtatgacg                                                                                                        <  1:224813‑M1/301‑194 (MQ=255)`  
                                                                                                                                                                                                  `aCTGGCTGACATTGCATCCACTAAATTATCCGATGATGCACGTGTGGCAAACCAAACTCAAAAGCTTTCCAAATCCTTTTAGTAATCAAACAGGAGAAAACAATATGatcgtttctgaaatagaagataacgcccgcttagagagcgtcactaagttccactgcggggttatctacgactagtcaaccgctgagtacgtaagctaccgtccgagtgacttcggtgcgtatctggatgcgctggaagccgaggttgcacgaggcggtcttattgtgttccacaacggtcacaagtatgacgt                                                                                                       <  1:248019‑M1/301‑195 (MQ=255)`  
                                                                                                                                                                                                     `ggCTGACAGTGCATCCACTAAAGTTACCGAGGCTGCCCGTGTGGCAAACCAAGCTCAACAGCTTTCCAAATTCTTTGAGTAATCAAACAGGAGAAACCATTATGatcgtttctgacatcgaagctaacgccctcttagagagcgtcactaagttccactgcggggttatctacgactactccaccgctgagtacgtaagctaccgtccgagtgacttcggtgcgtatctggatgcgctggaagccgaggttgcacgaggcggtcttattgtgttccacaacggtcacaagtatgactgtct                                                                                                    >  1:385687‑M1/1‑104 (MQ=255)`  
                                                                                                                                                                                                        `aagcagtGCATCCACTAAAGTTACCGCGGCTGCCCGTGTGGCAAACCAAGCTCAACAGCTTTCCAAATTCTAGGAGTAATCAAACAGGAGAAATCAATATGatcgtttctgacatcgaagctaacgccctcttagagagcgtcactaagttccactgcggggttatctacgactactccaccgctgagtacgtaagctaccgtccaagtgacttcggtgcgtatctggatgcgctggaagccgaggttgcacgaggcggtcttattgtgttccacaacggtcacaagtatgacgttcctgc                                                                                                 <  1:358969‑M1/298‑201 (MQ=255)`  
                                                                                                                                                                                                          `gtgtataagagacagaagttaccgaggctgccCGGGTGGCAAACCAAGCTCAACAGCTTTCCAAAGTCTTTGAGTAATCAAACAGGAGAAACCATTATGatcgtttctgacatcgaagctaacgccctcttagagagcgtcactaagttccactgcggggttatctacgactactccaccgctgagtacgtaagctaccgtccgagtgacttcggtgcgtatctggatgcgctggaagccgaggttgcacgaggcggtcttattgtgttccacaacggtcacaagtatgacgttcctgcat                                                                                               <  1:46967‑M1/286‑203 (MQ=255)`  
                                                                                                                                                                                                          `caggtgcATCCACTAAAGTTACCGAGGCTGCCCGTGTGGCAAACCAAGCTCAACAGCTTTCCAAATTCTTTGAGTAATCAAACAGGAGAAACCATTATGatcgtttctgacatcgaagttaacgccctcttagagagcgtcactaagttccactgctgggttatctacgactactccaccgctgagtacgtaagctaccgtccgagtgacttcggtgcgtatctggatgcgctggaagccgaggttgcacgagtcggtcttattgtgttccacaacggtcacaagtatgacgttcctgcat                                                                                               <  1:190587‑M1/298‑203 (MQ=255)`  
                                                                                                                                                                                                          `aaaaagacagccctaaagtgacagaGGCTGCACGAGTGGCAAACCAATCTCAACAGCTTTCCAAATTCTTTGCGTAATCAAACAGGGGAAGTCATTATAatcgtttctgacatcgaggctaacgccctcttagagagcgtcactaagttccactgcggggttatctacgactactccaccgctgagtacgtaagctaccgtccgagtgacttcggtgcgtatctggatgcgctggaagccgaggttgcacgaggcggtcttattgtgttccacaacggtcacaagtatgacgttcctgcat                                                                                               <  1:302328‑M1/289‑203 (MQ=255)`  
                                                                                                                                                                                                          `aCAGTGCATCCACTAAAGTTACCGAGGCTGCCCGTGTGGCAAACCAAGCTCAACAGCTTTCCAAATTCTTTGAGTAATCAAACAGGAGAAACCATTATGatcgtttctgacatcgaagctaacgccctcttagagagcgtcactaagttccactgcggggttatctacgactactccaccgctgagtacgtaagctaccgtccgagtgacttcggtgcgtatctggatgcgctggaagccgaggttgcacgaggcggtcttattgtgttccacaacggtcacaagtatgacgttcctgcat                                                                                               <  1:67028‑M1/301‑203 (MQ=255)`  
                                                                                                                                                                                                           `cAGTGCATCCACTAAAGTTACCGAGGCTGCCCGTGTGACAAACCAAGCTCAACAGCTTTCCAAATTCTTTGAGTAATCAAACAGGAGAAACCATTATGatcgtttctgacatcgcagctaacgccctcttagagagcggcactaagttccactgcggggttatctacgactactccaccgctgagtacgtaagctaccgtccgagtgacttcggtgcgtatctggatgcgctggaagccgcggttgcacgaggccgtcttattgttttccccaacggtcgcaagtatgacgttccttcatt                                                                                              >  1:101400‑M1/1‑98 (MQ=255)`  
                                                                                                                                                                                                                   `ccACTAAAGTTACCGAGGCTGCCCGTGTGGCAAACCAAGCTCAACAGCTTTCCAAATTCTTTGAGTAATCAAACAGGAGAAACCATTATGatcgtttctgacatcgaagctaacgccctcttagagagcgtcactaagttccactgcggggttatctacgactactccaccgctgagtacgtaagctaccgtccgagtgacttcggtgcgtatctggatgcgctggaagccgaggttgcacgaggcggtcttattgtgttccacaacggtcacaagtatgacgttcctgcattgaccaaac                                                                                      >  1:7248‑M1/1‑90 (MQ=255)`  
                                                                                                                                                                                                                                       `gCCCGGGTGGCAAACCAAGCGTAACAGCTTGCCAAATTCTTGGAGTAATCAAACAGGAGAAACCATTATGatcgtttctgacatcgaagctaacgccctcttagagagcgtcactaagttccactgcggggttatctgcgactactccaccgctgagtacgtaagctaccgtccgagtgactgcggtgcgtatctggatgcgctggaagccgaggttgcacgaggcggtcttattgtgttccacaacggtcacaagtatgacgttcctgcattgaccaaactggcaaagttgcaattgaac                                                                  <  1:255834‑M1/301‑232 (MQ=255)`  
                                                                                                                                                                                                                                        `cccGTGTGGCAAACCAAGCTCAACAGCTTTCCAAATTCTTTGAGTAATCAAACAGGAGAAACCATTATGatcgtttctgacatcgaagctaacgccctcttagagagcgtcactaagttccactgcggggttatctacgactactccaccgctgagtacgtaagctaccgtccgagtgacttcggtgcgtatctggatgcgctggaagccgaggttgcacgaggcggtcttattgtgttccacaacggtcacaagtatgacgttcctgcattgaccaaactggcaaagttgcaattgaacc                                                                 >  1:376270‑M1/1‑69 (MQ=255)`  
                                                                                                                                                                                                                                             `gtgGCAAACCAAGCTCAACAGCTTTCCAAATTCTTTGAGTAATCAAACAGGAGAAATCAATATGatcgtttctgacatcgaagctaacgccctcttagagagcgtcactaagttccactgcggggttatctacgactactccaccgctgagtacgtaagctaccgtccgagtgacttcggtgcgtatctggatgcgctggaatccgaggttgcacgaggcggtcttattgtgctccacaacggtcacaagtatgacgttcctgcattgaccaaactggcaaagttgcaattgaaccgagag                                                            >  1:447931‑M1/1‑64 (MQ=255)`  
                                                                                                                                                                                                                                                  `aaaCCAAGCTCAACAGCTTTCCAAATTCTTTGAGTAATCAAACAGGAGAAACCATTATGatcgtttctgacatcgaagctaacgccctcttagagagcgtcactcagttccactgcggggttatctacgactactccaccgctgagtacgtaagctaccgtccgagttacttcggtgcgtatatggatgcgctggaatacgcggttgcacgaggcgctcttattgtgttccacaacagtcaccagtataccgttcctgccttcaccaacctgacacatattcatttcttttccttatacca                                                       >  1:280951‑M1/1‑59 (MQ=255)`  
                                                                                                                                                                                                                                                    `gatgggtataagagacagccaaattctttgagtaatcaAACAGGAGAAACCATTATGatcgtttctgacatcgaagctaacgccctcttagagagcgtcactaagttccactgcggggttatctacgactactccaccgctgagtacgtaagctaccgtccgagtgacttcggtgcgtatctggatgcgctggaagccgaggttgcacgaggcggtcttattgtgttccacaacggtcacaagtatgacgttcctgcattgaccaaactggcaaagttgcaattgaaccgagagttccacc                                                     <  1:309800‑M1/283‑245 (MQ=255)`  
                                                                                                                                                                                                                                                     `ccAAGCTCAACAGCTTTCCAAATTCTTTGAGTAATCAAACAGGAGAAACCATTATGctcgtttctgacatcgaagctaacgccctcttagagagcgtcactaagttccactgcggggttatctacgactactccaccgctgagtacgtaagctaccgtccgagtgacttcggtgcgtatctggatgcgctggaagccgaggttgcacgaggcggtcttattgtgttccacaacggtcacaagtatgacgttcctgcattgaccaaactggcaaagttgcaattgaacagagagttccacct                                                    <  1:179487‑M1/301‑246 (MQ=255)`  
                                                                                                                                                                                                                                                                           `ttCTTTGAGTAATCAAACAGGAGAAACCATTATGatcgtttctgacatcgaagctaacgccctcttagagagcgtcactaagttccactgcggggttatctacgactactccaccgctgagtacgtaagctaccgtccgagtgacttcggtgcgtatctgtatgcgctggaagccgaggttgcacgaggcggtcttattgttttctacaccgttcacaagtatcacgttcctgcatttaccaaattgtcaacgttgcaattgaacctacagttcaaccttcctcgtgagaactgtatctac                              >  1:284121‑M1/1‑34 (MQ=255)`  
                                                                                                                                                                                                                                                                            `tCTTTGAGTAATCAAACAGGAGAAACCATTATGatcgtttctgacatggaagctaacgccctcgtagagagcgtcactaagtgccactgcggggttatctacgactactccaccgctgagtacgtaagctaccgtccgagtgacttcggtgcgtatctggatgcgctggaagccgaggttgcacgaggcggtcttattgtgttccacaacggtcacaagtatgacgttcctgcattgaccaaactggcaaagttgcaattgaaccgagagttccaccttcctcgtgagaactgtattgaca                             <  1:434779‑M1/301‑269 (MQ=255)`  
                                                                                                                                                                                                                                                                             `cTTTGAGTAATCACACAGGAGAAACCATTATGatcgtttctgacatcgaagctaacgccctcttagagagcgtcactaagttccactgcggggttatctacgactactccaccgctgagtacgtaagctaccgtccgagtgacttcggtgcgtatctggatgcgctggaagccgaggttgcacgaggcggtcttattgtgttccacaacggtcacaagtatgacgttcctgcattgaccaaactggcaaagttgcaattgaaccgagagttccaccttcctcgtgagaactgtattgacac                            <  1:362860‑M1/301‑270 (MQ=255)`  
                                                                                                                                                                                                                                                                                     `agacagctgggagaaaccatTATGatcgtttctgaaatcgaagctaacgccctcttagagagcgtcactaagttccactgcggggttatctacgactactccaccgatgagtacgtaagctaccgtccgagtgacttcggtgcgtatctggatgcgctggaagccgaggttgcacgaggcggtcttattgtgttccacaacggtcacaagtatgacgttcctgcattgaccaaaccggcaaagttgcaattgaaccgagagttccaccttcctcgtgagaactgtattgacacccttgtgt                    <  1:493244‑M1/292‑278 (MQ=255)`  
                                                                                                                                                                                                                                                                                     `aaTCAAACAGTAGAAACCATTATCatcgtttctgacatcgaagctaaggccaccttagagagcgtcatgaagttccactgcgggtttatctacgactactccaccgctgagtacgtaagctaccgtccgagtgacttcggtgcgtatctggatgcgctggaagccgaggttgcacgaggcggtcttattgtgttccacaacggtcacaagtatgacgttcctgcattgaccaaactggcaaagttgcaattgaaccgagagttccaccttcctcgtgagaactgtattgacacccttgtgt                    <  1:11644‑M1/301‑278 (MQ=255)`  
                                                                                                                                                                                                                                                                                     `aaTCAAACAGGAGAAATCAATAGGatcgtttctgacattgaagctaacgccctcttagagagcgtcattaagttccactgcggggttatctacgactacgccaccgctgagtacgtaagctaccgtccgagtgacttcggtgcgtatctggatgcgctggaagccgaggttgcacgatgcggtcttattgtgttccacaacggtcacaagtatgacgttcctgcattgaccaaactggcaaagttgcaattgaaccgagagttccaccttcctcgtgagaactgtattgacacccttgtgt                    <  1:106238‑M1/301‑278 (MQ=255)`  
                                                                                                                                                                                                                                                                                     `aaTCAAACAGGAGAAACCATTATGatcgtttctgacatcgaagctaacgccctcttagagagcgtcactaagttccactgcggggttatctacgactactccaccgctgagtacgtaagctaccgtccgagtgacttcggtgcgtatctggatgcgctggaagccgaggttgcacgaggcggtcttattgtgttccacaacggtcacaagtatgacgttcctgcattgaccaaactggcaaagttgcaattgaaccgagagttccaccttcctcgtgagcactgtattgacaaccttgtgt                    >  1:210679‑M1/1‑24 (MQ=255)`  
                                                                                                                                                                                                                                                                                      `aTCAAACAGGAGAAACCATTATGatcgtttcttacatcgaagctaacgccctcttagagagcgtcactaagttccactgcggggttatctacgactactccaccgctgagtacgtaagctaccgtccgagtgacttcggtgcgtatctggatgcgctggaagccgaggttgcacgaggcggtcttattgtgttccacaacggtcacaagtatgacgttcctgcattgaccaaactggcaaagttgcaattgaaccgagagttccaccttcctcgtgagaactgtattgacaccctgtctct                   >  1:208092‑M1/1‑23 (MQ=255)`  
                                                                                                                                                                                                                                                                                      `aTCAAACAGGAGAAACCATTATGatcgtttctgacatcgaagctaacgccctcttagagtgcgtcactaagttccactgcggggttatctacgactactacaccgctgagtacgtaagctaccgtccgagtgacttcggtgcgtatctggatgcgctggaagccgaggttgcacgaggcgggcttattgtgttgcacaacggtcacaagtatgacgttcctgcattgaccaaactggcaaagttgcaattgaaccgagagttcaaccttcctagtgagaaatgtattgacacccttatgtt                   >  1:326246‑M1/1‑23 (MQ=255)`  
                                                                                                                                                                                                                                                                                                     `ccATTATGatcgtttctgacatcgaagctaacgccctcttagagagcgtcactaagttccactgcggggttatctacgactactcaaccgctgagtacgtaagctaccgtccgagtgacttcggtgcgtatctggatgcgctggaagccgaggttgcacgaggcggtcttattgtgttccacaacggtcacaagtctgacgttcctgcattgaccaaactggcaaagttgcaattgaaccgagagttccaccttcctcgtgagaactgtattgacacccttgtgttgtcacgtttgattac    >  1:77719‑M1/1‑8 (MQ=255)`  
                                                                                                                                                                                                                                                                                                       `aTTATGatcgtttctgacatcgaagctaacgccctcttagagagcgtcactaagttccactgcggggttatctacgactactccaccgctgagtacgtaagctaccgtccgagtgacttcggtgcgtatctggatgcgctggaagccgaggttgcacgaggcggtcttattgtgttccacaacggtcacaagtatgacgttcctgcattgaccaaactggcaaagttgcaattgaaccgagagttccaccttcctcgtgagaactgtattgacctgtctcttatacacatctccgagcccc  >  1:221176‑M1/1‑6 (MQ=255)`  

                                                                                                                                                                                                                                                                                                            `|`                                                                                                                                                                                                                                                                                                         
`CAGACGACTACGAGAAGTTTCGCTGGAGAGTCCCATTCTAATACGACTCACTAAAGGAGACACACCATGTTCAAACTGATTAAGAAGTTAGGCCAACTGCTGGTTCGTATGTACAACGTGGAAGCCAAGCGACTGAACGATGAGGCTCGTAAAGAGGCCACACAGTCACGCGCTCTGGCGATTCGCTCCAACGAACTGGCTGACAGTGCATCCACTAAAGTTACCGAGGCTGCCCGTGTGGCAAACCAAGCTCAACAGCTTTCCAAATTCTTTGAGTAATCAAACAGGAGAAACCATTATGTCTAACGTAGCTGAAACTATCCGTCTATCCGATACAGCTGACCAGTGGAACCGTCGAGTCCACATCAACGTTCGCAACGGTAAGGCGACTATGGTTTACCGCTGGAAGGACTCTAAGTCCTCTAAGAATCACACTCAGCGTATGACGTTGACAGATGAGCAAGCACTGCGTCTGGTCAATGCGCTTACCAAAGCTGCCGTGACAGCAATTCATGAAGCTGGTCGCGTCAATGAAGCTATGGCTATCCTCGACAAGATTGATAACTAAGAGTGGTATCCTCAAGGTCGCCAAAGTG  >  NC_001604.1/13286‑13881`

  

Alignment Legend

Aligned base mismatch/match (shaded by quality score): `ATCG/ATCG` `< 3 ≤` `ATCG/ATCG` `< 9 ≤` `ATCG/ATCG` `< 19 ≤` `ATCG/ATCG` `< 34 ≤` `ATCG/ATCG` `< 38 ≤` `ATCG/ATCG`

Unaligned base: `atcg`    Masked matching base: `atcg`    Alignment gap: `‑`    Deleted base: `‑`

Reads not counted as support for junction

read\_name Not counted due to insufficient overlap past the breakpoint.

read\_name Not counted due to not crossing MOB target site duplication.
